# The effects of stand spatial structure on the aboveground biomass allocation in Chinese fir (*Cunninghamia lanceolata*) plantations

**DOI:** 10.3389/fpls.2025.1599094

**Published:** 2025-06-19

**Authors:** Xiang Huang, Yao Zhang, Jianwei Geng, Xiangyu Chen, Zhihui Yu, Shuhan Yu, Kunyong Yu, Fan Wang, Jian Liu

**Affiliations:** ^1^ College of Forestry, Fujian Agriculture and Forestry University, Fuzhou, China; ^2^ University Key Lab for Geomatics Technology & Optimize Resource Utilization in Fujian Province, Fujian Agriculture and Forestry University, Fuzhou, China; ^3^ College of JunCao Science and Ecology (College of Carbon Neutrality), Fujian Agriculture and Forestry University, Fuzhou, China; ^4^ College of Landscape Architecture and Art, Fujian Agriculture and Forestry University, Fuzhou, China

**Keywords:** UAV lidar, Chinese fir, spatial structure, biomass, distribution pattern

## Abstract

**Introduction:**

Chinese fir (*Cunninghamia lanceolata*) is the fastest-growing timber species in China. investigating its spatial structure and influence on aboveground biomass allocation is crucial for understanding its adaptability to environmental conditions, enhancing carbon sequestration, and maintaining forest ecosystem stability.

**Methods:**

In this study, airborne LiDAR technology was used to derive forest structural metrics, and weighted Voronoi diagrams were constructed to extract spatial configuration metrics. Biomass models for different components of Chinese fir were developed using 20 harvested trees, and stem mass fraction (SMF), branch mass fraction (BMF), and leaf mass fraction (FMF) were calculated. Path analysis quantified the effects of stand structure variables on biomass allocation among different organs.

**Results:**

The openness ratio (OP), angle competition index (UCI), forest layer index (S), and openness (K) were identified as the primary spatial structural factors influencing aboveground biomass allocation. Stem biomass accumulation is maximized when 0.75 < OP ≤ 1, 0 < UCI ≤ 0.25, 0 < S ≤ 0.25, and 0.4 < K ≤ 0.5, with SMF reaching its highest value. Branch biomass peaks when 0.5 < OP ≤ 0.75, 0 < UCI ≤ 0.25, 0.75 < S ≤ 1, and 0.4 < K ≤ 0.5, maximizing BMF. Leaf biomass is highest when 0 < OP ≤ 0.25, 0.5 < UCI ≤ 0.75, 0.5 < S ≤ 0.75, and 0.2 < K ≤ 0.3, leading to the maximum FMF.

**Discussion:**

The results of this study not only reveal the survival strategy of Chinese fir in environmental change, but also provide a theoretical basis for understanding ecosystem carbon sequestration and sustainable management of Chinese fir plantations.

## 1 Introduction

Biomass is a key indicator of forest ecosystem health, structure, and function (Khan et al., 2021; Saarela et al., 2022; Salunkhe et al., 2018). Its distribution among different organs of forest trees is known as biomass allocation. Understanding how plants allocate resources is crucial in forest ecology (Chen et al., 2021; Vasseur et al., 2023; Weiner, 2004). Generally, biomass allocation can be studied in two ways: by analyzing biomass fractions, which examine the proportion of individual organs relative to total biomass, and by investigating the quantitative relationships between different organ biomass measurements (Poorter and Nagel, 2000).

In forest ecosystems, plants exhibit varying biomass allocation patterns depending on environmental conditions, a phenomenon known as the plasticity of biomass allocation. This plasticity determines a plant’s ability to access heterogeneous resources (SEBASTIA, 2007; Wu et al., 2022). Resource allocation among different organs ensures survival by optimizing biomass distribution patterns, reflecting a plant’s adaptability to external conditions (Dolezal et al., 2021; Yan et al., 2016). Numerous factors influence biomass distribution. Climatic conditions play a key role, as reduced rainfall leads to drought, inhibiting plant growth by decreasing soil moisture and nutrients (Schimel, 2018). To cope with drought stress, plants increase their root–shoot ratio to enhance water and nutrient uptake (Tang et al., 2024). Conversely, increased rainfall promotes aboveground biomass allocation, maximizing light energy capture (Zhang and Xi, 2021). Other factors, including soil pollution (Delerue et al., 2022), anthropogenic crop rotation (Oliveira et al., 2018), and intraspecific competition (Wertz et al., 2020), also affect plant biomass distribution. Therefore, examining these factors of plant biomass can help us better understand the coping strategies of plants in different environments and the carbon cycle and carbon accumulation processes in forest ecosystems.

Forest structures are composed of spatial and non-spatial structures that determine the stability, direction of succession and management space of forest stands (Hui et al., 2019). Among them, the forest stand spatial structure characterizes the distributional arrangement and relative positioning of trees, shaping tree competition and resource utilization (Pang et al., 2024). A well-structured stand optimizes space, water, soil, and light resources, creating favorable growth conditions (Fang et al., 2021; He et al., 2022). As spatial arrangement is a manageable factor, it is often used to guide harvesting and regeneration. Spatial structure parameters inform harvesting optimization models that enhance tree distribution suitability (Chen et al., 2023; Zhang et al., 2018, 2022). Stand spatial structure also affects tree growth and understory vegetation diversity. Cao et al. (2020a) found that horizontal stand structure had a greater influence on understory diversity than vertical structure or tree competition. Similarly, Zhu et al. (2018) identified mixing angle and horizontal tree distribution as primary factors affecting understory shrub species diversity. Given its importance, understanding forest stand geometry is essential for sustainable forest management and ecosystem stability. Analyzing stand spatial structure requires establishing spatial relationships among individual trees. Attributes such as tree position must be considered when developing weighted Voronoi diagrams to ensure an accurate representation of tree relationships (Feng et al., 2014; Li et al., 2015; Liu et al., 2023). While these diagrams segment structural units, manual location data collection in sample plots remains labor-intensive, requiring considerable resources. At present, with the rapid development and application of airborne lidar technology in forestry, it provides an efficient technical means for the reconstruction of three-dimensional structure of forests. By actively emitting laser pulses and receiving reflected signals, UAV-LiDAR can quickly obtain high-precision 3D point cloud data, and realize accurate inversion of forest parameters by using single wood segmentation and point cloud data processing technology (Fisher et al., 2020). Compared with manual surveying, UAVs not only greatly improve the efficiency of data collection, but also cover complex terrain and a wide range of sample plots, reduce human error, and reduce the cost and risk of field operations (Wang et al., 2019). In terms of applied research, Chen et al. (2025) used airborne Lidar and field survey data to extract the position and height of individual trees of multiple tree species according to CHM and NPC, and then constructed a tree height-DBH model of forest trees, which provides a scientific basis for the application of lidar in forest ecosystems. Ye et al. (2025), based on XGBoost and PLSR machine learning algorithms, combined with 96 canopy structure indexes extracted by UAV-LiDAR, achieved high-precision estimation of eucalyptus DBH (R²=0.829) and biomass (R²=0.903), and confirmed that LiDAR height and voxel indexes are the key characteristic parameters for accurate measurement of forest carbon sinks. Xu et al. (2024) reconstruct realistic 3D forest scenes based on UAV-LiDAR point cloud data, and propose a 3D modeling method for complex tree-shrub-grass forest scenes, considering the hierarchical structure of trees, shrubs and grasses in the forest, with a single tree segmentation accuracy of 87.3%, shrub segmentation accuracy of 60%, and grassland height accuracy evaluation of RMSE<0.15m, which provides technical support for meeting the needs of large-scale forest scene modeling. The above results show that UAV-LiDAR can not only efficiently obtain the single-tree scale structural parameters, but also provide a theoretical basis for the extraction of stand spatial structure parameters, and can use UAV data to obtain the location information and basic attribute parameters of forest trees, and combine forest tree attributes with spatial attributes to determine the spatial structure units of forest trees by constructing a weighted Voronoi diagram, so as to realize the rapid calculation of spatial structure parameters in the sample plot. The spatial structure of forest stands is closely linked to forest carbon pools (Li et al., 2023), influencing competition, growth potential, and the development of adjacent trees (Weng et al., 2019). Stand spatial structure directly affects forest quality and carbon sequestration capacity, yet its impact on biomass distribution remains unclear. Evaluating how tree spatial arrangement influences biomass distribution can improve carbon storage optimization and ecosystem resilience in forest management.

Chinese fir (*Cunninghamia lanceolata*) is a vital timber species in southern China, known for its rapid growth, strong environmental adaptability, high timber yield, and insect resistance. Its extensive applications make it a preferred species for carbon-neutral forest initiatives. Investigating how stand spatial structure affects the distribution of aboveground biomass (AGB) in Chinese fir is crucial for understanding its adaptation strategies and enhancing carbon sequestration in plantations. This study examines Chinese fir plantations using UAV LiDAR data to extract apex height, tree height, crown width, and DBH. A weighted Voronoi diagram was constructed to compute spatial structure parameters across six stands, including size ratio, angle competition index, forest layer index, openness ratio, openness, and uniform angle. A biomass model developed from 20 felled trees was integrated with forest parameters derived from point cloud data to estimate biomass for each organ. The proportion of trunk, branch, and leaf biomass in total AGB was then calculated. Path analysis quantified the effects of various structural indices on biomass allocation in different organs of Chinese fir.

## 2 Methodologies and materials

### 2.1 Study area

**Figure 1:**
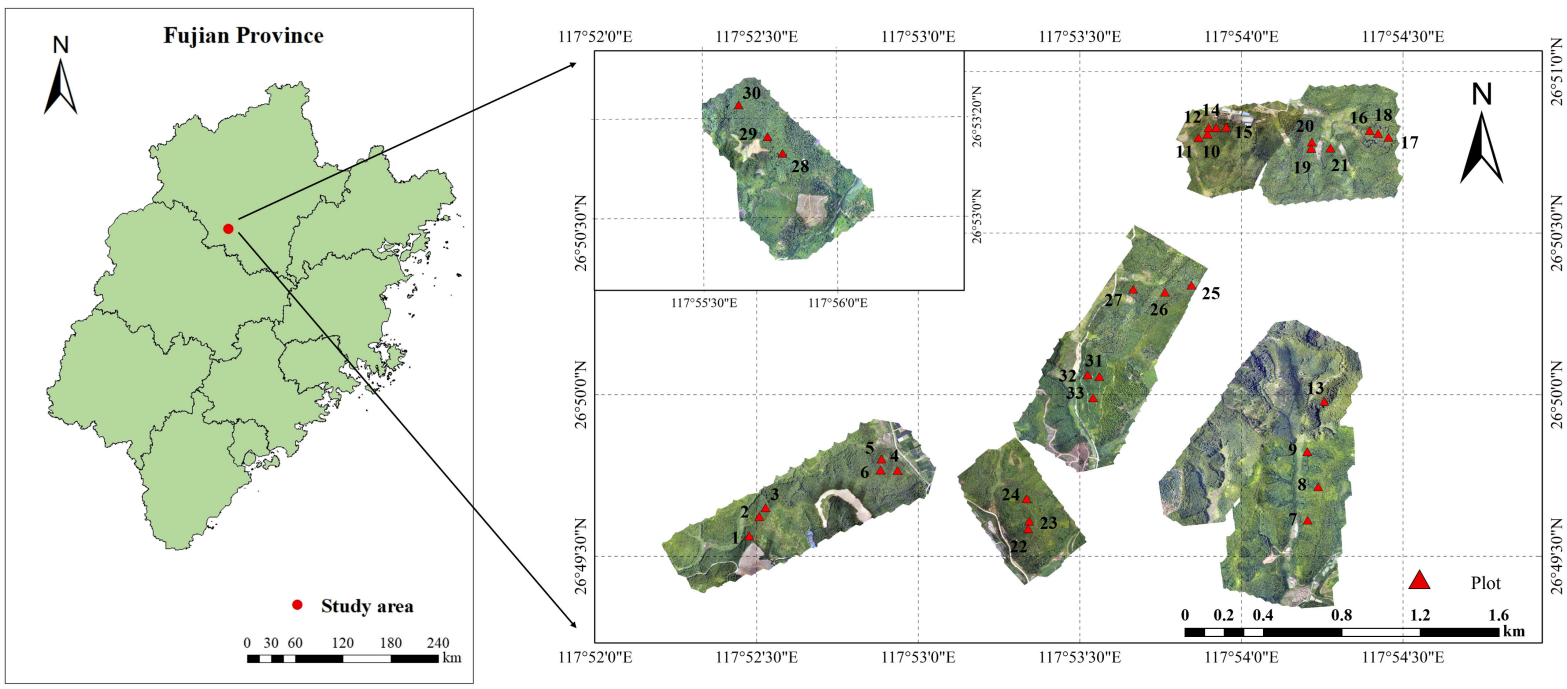
Overview map of the study area.

The study area is situated within the Yangkou State-owned Forest Farm in Shunchang County, Nanping City, Fujian Province, located at 117°29′~118°14′ E longitude, 26°38′~27°12′ N latitude, 100–800 m above sea level, 15–30° slope (**Figure 1**). The area has a central subtropical oceanic climate characterized by warm, humid conditions. The average annual temperature is approximately 20°C, with an annual precipitation of 2144.2 mm, 1740 h of sunshine, and a frost-free period of 289 d. The soil is primarily deep, fertile, mountainous red soil. As one of the core production areas for Chinese fir in southern China, the region also hosts Masson pine (*Pinus massoniana*), eucalyptus (*Eucalyptus robusta*), and Moso bamboo (*Phyllostachys heterocycla*).

### 2.2 Dataset acquisition

#### 2.2.1 Ground data collection

In this study, 33 sampling plots measuring 25.82 × 25.82 m were set up in Yangkou Forest Farm in August 2022, and three young forests, six middle-aged forests, six near-ripe forests, six mature forests, and 12 overripe forests were selected to ensure the universality of the results. Survey information from the sample plots, including the average tree height, average crown width, average DBH, and other data, was procured. The survey results are shown in **Table 1**. The results are shown as the average results ± standard deviations for each age group. Approximately 10 trees comprising a total of 353 trees were arbitrarily chosen from each sample plot for RTK positioning (**Table 2**). This data served to validate UAV measurement accuracy and construct the DBH model.

**Table 1:** Plot survey information form.

Age Group	Tree height /m	CW /m	DBH /cm	Stand Density /Trees/ha	Canopy Density	Slope/°	Altitude/m
Young forest	9.1 ± 0.8	2.4 ± 0.2	12.2 ± 1.5	2533.7 ± 320.6	0.8 ± 0.02	23.0 ± 3.4	220.7 ± 8.10
Middle-aged forest	13.2 ± 1.8	2.5 ± 0.6	14.6 ± 2.3	2006.5 ± 950.2	0.7 ± 0.05	27.3 ± 3.5	227.8 ± 47.7
Near-ripe forest	16.4 ± 1.2	2.6 ± 0.6	17.9 ± 1.5	2088.9 ± 265.9	0.8 ± 0.10	24.1 ± 3.1	215.5 ± 36.6
Mature forest	17.7 ± 1.1	2.8 ± 0.3	21.1 ± 2.5	1256.8 ± 507.5	0.7 ± 0.10	24.5 ± 4.8	233.5 ± 30.9
Overripe forest	22.9 ± 3.9	3.0 ± 0.7	21.4 ± 6.0	1279.4 ± 522.5	0.7 ± 0.10	29.2 ± 5.0	237.3 ± 50.9

CW is the crown width of the trees and DBH is the diameter at breast height of the trees.

**Table 2:** Sample tree information form.

Sample Plot No.	Number of sample trees	Average tree height/m	Average CW/m	Average DBH/cm	Sample Plot No.	Number of sample trees	Average tree height/m	Average CW/m	Average DBH/cm
1	10	11.8 ± 0.7	2.1 ± 0.2	14.9 ± 1.3	18	10	21.3 ± 0.9	3.3 ± 0.2	33.5 ± 2.9
2	10	12.5 ± 0.6	2.3 ± 0.3	15.1 ± 0.8	19	11	16.8 ± 1.0	2.4 ± 0.3	17.9 ± 1.8
3	10	17.6 ± 0.9	2.5 ± 0.1	21.2 ± 2.4	20	15	17.5 ± 1.1	2.1 ± 0.2	18.4 ± 1.4
4	10	19.3 ± 0.3	2.6 ± 0.3	18.0 ± 3.0	21	12	19.3 ± 0.8	2.9 ± 0.3	21.4 ± 2.0
5	10	18.6 ± 1.2	2.6 ± 0.1	21.0 ± 2.8	22	10	15.0 ± 1.1	2.5 ± 0.3	17.6 ± 1.9
6	12	17.6 ± 0.8	3.0 ± 0.2	19.7 ± 2.2	23	11	14.0 ± 0.9	1.8 ± 0.1	17.7 ± 1.7
7	11	16.2 ± 0.6	2.3 ± 0.3	17.4 ± 2.3	24	14	17.4 ± 0.5	3.0 ± 0.1	20.4 ± 1.4
8	10	13.0 ± 1.3	2.9 ± 0.2	14.1 ± 2.1	25	11	19.9 ± 0.8	3.4 ± 0.1	26.7 ± 2.1
9	10	17.1 ± 0.8	1.9 ± 0.1	20.3 ± 2.8	26	10	17.7 ± 0.7	2.8 ± 0.2	20.8 ± 2.3
10	9	12.5 ± 0.5	2.2 ± 0.1	17.6 ± 1.3	27	10	19.3 ± 0.4	3.5 ± 0.3	22.8 ± 1.7
11	10	13.3 ± 0.8	2.1 ± 0.2	16.6 ± 1.5	28	10	23.4 ± 0.6	3.4 ± 0.3	32.1 ± 2.8
12	11	12.7 ± 0.5	2.2 ± 0.2	17.1 ± 1.3	29	12	19.1 ± 0.8	2.6 ± 0.1	28.7 ± 2.5
13	12	18.5 ± 1.3	2.4 ± 0.7	27.2 ± 3.5	30	11	23.1 ± 03	3.0 ± 0.1	35.0 ± 1.7
14	10	24.3 ± 0.6	4.3 ± 0.3	40.5 ± 3.3	31	11	8.5 ± 0.5	1.8 ± 0.3	12.5 ± 1.5
15	8	20.8 ± 0.7	3.6 ± 0.2	39.7 ± 3.7	32	11	9.5 ± 0.5	1.9 ± 0.2	14.3 ± 1.3
16	8	24.8 ± 0.7	4.0 ± 0.5	27.6 ± 2.9	33	11	10.5 ± 0.2	2.1 ± 0.1	13.9 ± 0.7
17	12	20.8 ± 0.8	3.8 ± 0.2	27.1 ± 2.7					

#### 2.2.2 UAV data collection and pre-processing

**Figure 2:**
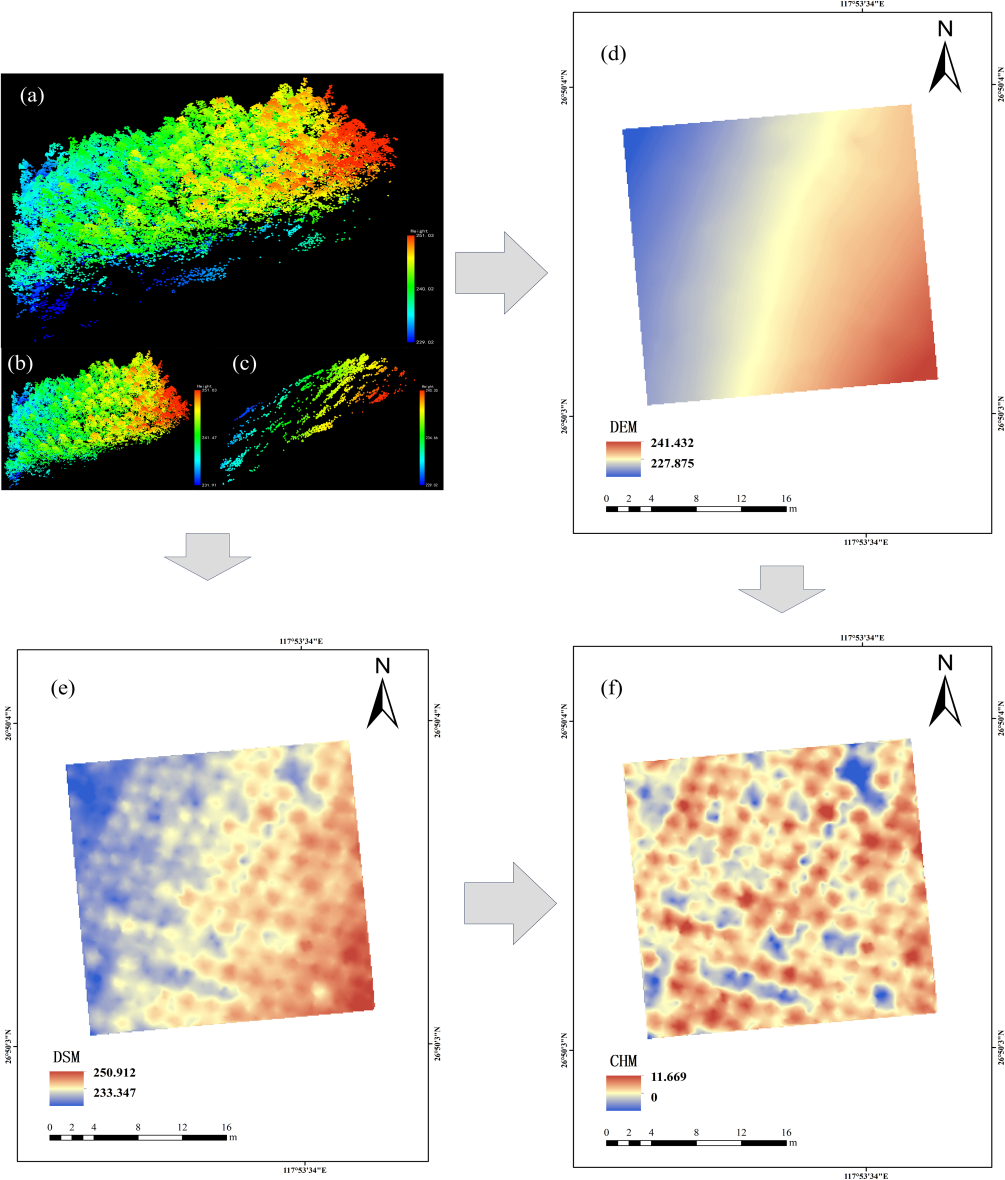
Point cloud data preprocessing, taking Y31 sample plot as an example. **(a)** sample site cloud data; **(b)** canopy point cloud of the sample plot; **(c)** Ground point cloud of the sample plot; **(d)** digital elevation models; **(e)** digital surface models; **(f)** Canopy height model.

The dense LiDAR point cloud dataset was acquired utilizing a Pegasus D500 UAV integrated with a HESAI XT32 sensor. The aerial survey employed a terrestrial flight pattern, maintaining an elevation of 150 m above ground level, with a velocity of 10 m/s, while the lateral overlap percentage of laser scanning was configured at 80%. The system operated in three-echo mode, and the laser classification was designated as CLASS1. The point cloud data were spliced using DJI Terra software. The LAS point cloud files were imported into Cloud Compare V2.13 software for preprocessing, and the obtained point cloud data were denoized. The denoised point cloud was separated using the cloth filtering algorithm (CSF). The ground point cloud was interpolated using kriging interpolation to create DEM (0.1 × 0.1 m). The non-ground point cloud interpolation was used to generate DSM (0.1 × 0.1m), and the CHM (0.1 × 0.1m) was obtained by subtracting the DSM and DEM of each sample plot (**Figure 2**).

### 2.3 Development of aboveground organ biomass model of Chinese fir

In this study, 20 representative Chinese fir were selected from the sample plots for harvesting (**Table 3**). Basic information on the harvested trees was recorded, and stratified harvesting was performed (Lin et al., 2019).

**Table 3:** Harvested wood biomass information sheet.

Tree NO.	Dry weight on the ground/kg	Dry weight of Stem/kg	Dry weight of branch/kg	Dry weight of leaf/kg	DBH/cm	H/m
1	23.42	16.07	1.82	5.53	12.4	8.4
2	21.78	14.55	1.79	5.44	11.8	9.2
3	24.2	16.51	1.98	5.71	12.8	9.6
4	26.25	18.12	2.14	5.99	13.2	11.4
5	58.49	44.61	4.95	8.93	16.5	13
6	60.37	47.09	5.01	8.27	16.7	14.1
7	58.82	43.78	5.51	9.53	15.8	16.7
8	63.53	46.5	7.09	9.94	17.2	17.1
9	66.43	50.12	6.88	9.43	17.8	18.1
10	77.56	59.33	7.91	10.32	20.7	19.7
11	83.95	65.22	8.31	10.42	21.6	19.9
12	86.61	66.1	9.06	11.45	23.5	19.5
13	96.54	71.76	14.02	10.76	23.2	19
14	101.79	76.8	12.66	12.33	25.1	18.8
15	128.86	103.02	15.03	10.81	28.8	19.4
16	172.04	131.87	26.4	13.77	29.3	20.3
17	188.89	151.3	25.28	12.31	31.8	23.1
18	203.25	162.61	27.61	13.03	32.5	26.2
19	208.16	170.74	26.07	11.35	33.8	23.4
20	220.29	176.45	29.71	14.13	35.7	24.1

To develop an AGB measurement model using tree height and DBH as independent variables, we evaluated nine commonly used correlations (**Table 4**) and selected the optimal model to estimate the biomass of aboveground organs. The biomass fractions of the stem, branches, and leaves were then determined by dividing their respective biomass by the total AGB, yielding the stem mass fraction (SMF), branch mass fraction (BMF), and leaf mass fraction (FMF).

**Table 4:** Biomass estimation model based on tree height and DBH.

Model NO.	Formula	Variable
1	W_1_=aH^b^	H
2	W_2_=aD^b^	D
3	W_3_=a(D^2^H)^b^	D^2^H
4	W_4_=a+b(D^2^H)	D^2^H
5	lnW_5_=a+blnD	D
6	lnW_6_=a+bD+cD^2^	D
7	lnW_7_=a+blnH	H
8	lnW_8_=a+bln(D^2^H)	D^2^H
9	lnW_9_=a+blnD+clnH	D, H

D is the diameter at breast height (DBH) of the trees, H is the height of the trees, and W is the biomass of the corresponding component of the trees.

### 2.4 Extraction of stand spatial structure parameters

#### 2.4.1 Individual tree segmentation

**Figure 3:**
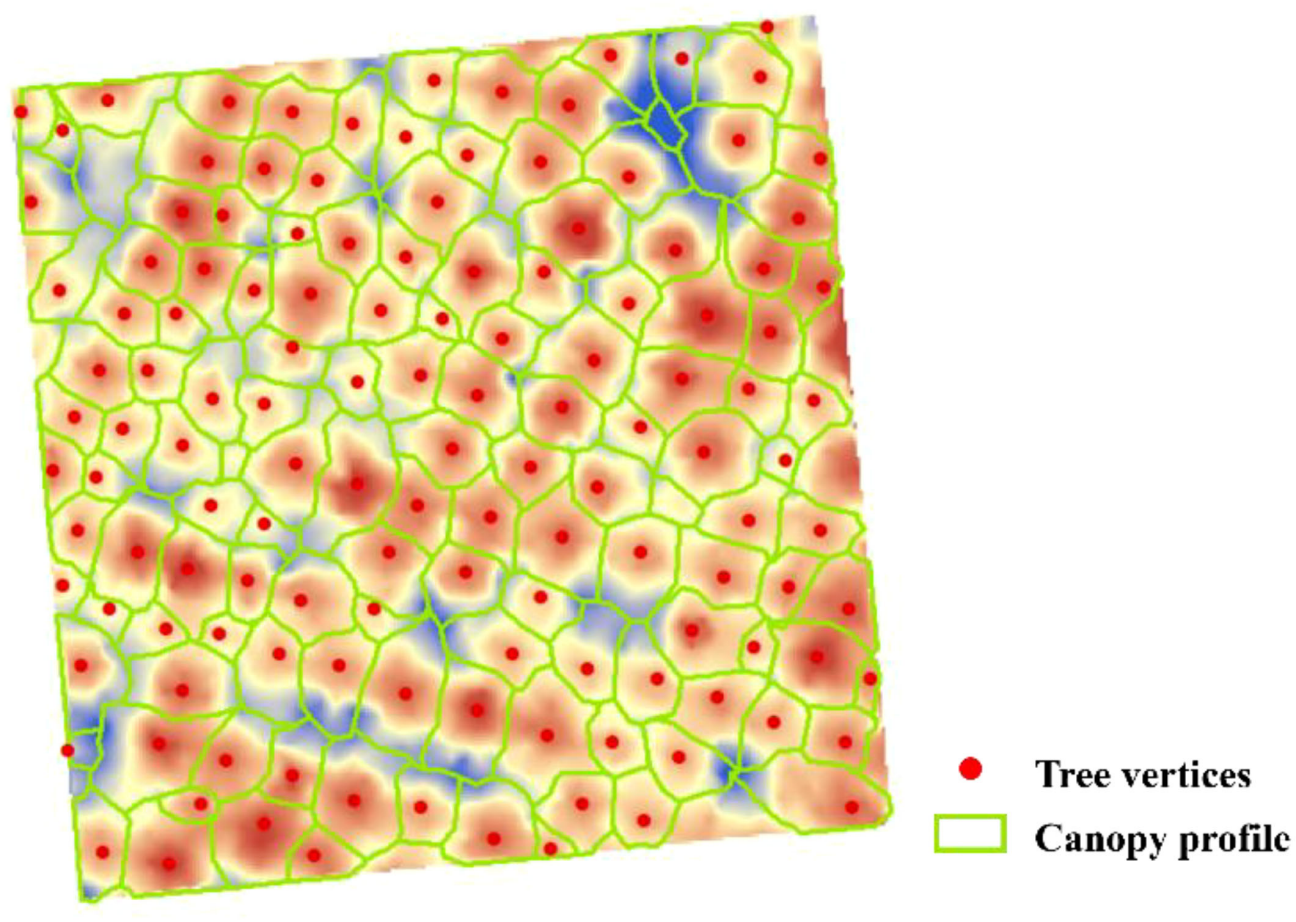
The results of tree vertex and canopy contour extraction based on local maxima and watershed methods, take the extraction results of Y31 sample plot as an example.

In this investigation, CHM was used for single-wood segmentation. Before dividing a single tree, the location of each tree was first detected to determine the position information of the vertex of the single tree. Then, the extracted tree vertex served as the seed point to segment and extract the crown of the single tree. In the tree vertex detection process, the local maximum value algorithm was utilized to determine the local maximum value in the raster image by continuously moving the fixed-size detection window and using it as a canopy vertex. Then, the watershed algorithm was used to reverse and invert the tree canopy, transforming the crown region into a drainage basin. The image was divided into several regions using the image segmentation algorithm of geomorphology, and segmentation of the tree crown was conducted through the best segmentation window (**Figure 3**). In this study, the detection rate (Equation 1), accuracy (Equation 2), and F-score (Equation 3) were used to evaluate the accuracy of the vertex extraction of a single tree and determine the ideal configuration of local maximum detection windows, which was calculated as follows:


(1) $$\begin{array}{c} r=\frac{TP}{TP+FN} \end{array}$$

(2) $$\begin{array}{c} P=\frac{TP}{TP+FP} \end{array}$$

(3) $$\begin{array}{c} F=\frac{2\left(r\times p\right)}{r+p} \end{array}$$

where TP denotes the successfully identified trees, FN signifies the missed trees in detection, FP indicates instances of trees incorrectly classified by the algorithm, r stands for the detection rate, p represents the detection precision, and the F-score reflects the detection accuracy.

Chinese fir is a coniferous tree with a conical crown shape and advantages over tree vertices. Therefore, the tree height was assigned to the corresponding CHM raster value for each detected tree vertex. Meanwhile, the average values of the north–south, and east–west widths of the canopy after single tree segmentation were calculated as the crown width. The measured tree height and crown width of 353 trees with RTK positioning in 33 sample plots were used to verify the accuracy of the UAV estimation after individual tree segmentation.

#### 2.4.2 Construction of DBH model of Chinese fir

Dense point clouds obtained from airborne lidar can accurately extract parameters such as tree height and crown width. Consequently, DBH can be indirectly obtained by establishing a model (Mao et al., 2023). In this study, the correlation between UAV-estimated tree height and crown width for 353 trees and their corresponding measured DBH was tested to determine whether there was a significant correlation. A UAV-based DBH estimation model for Chinese fir was constructed using multiple linear regression. To assess its accuracy, 70% of the 353 trees were used as the training set, while the remaining 30% served as the validation dataset.

#### 2.4.3 Extraction of stand spatial structure parameters

**Figure 4:**
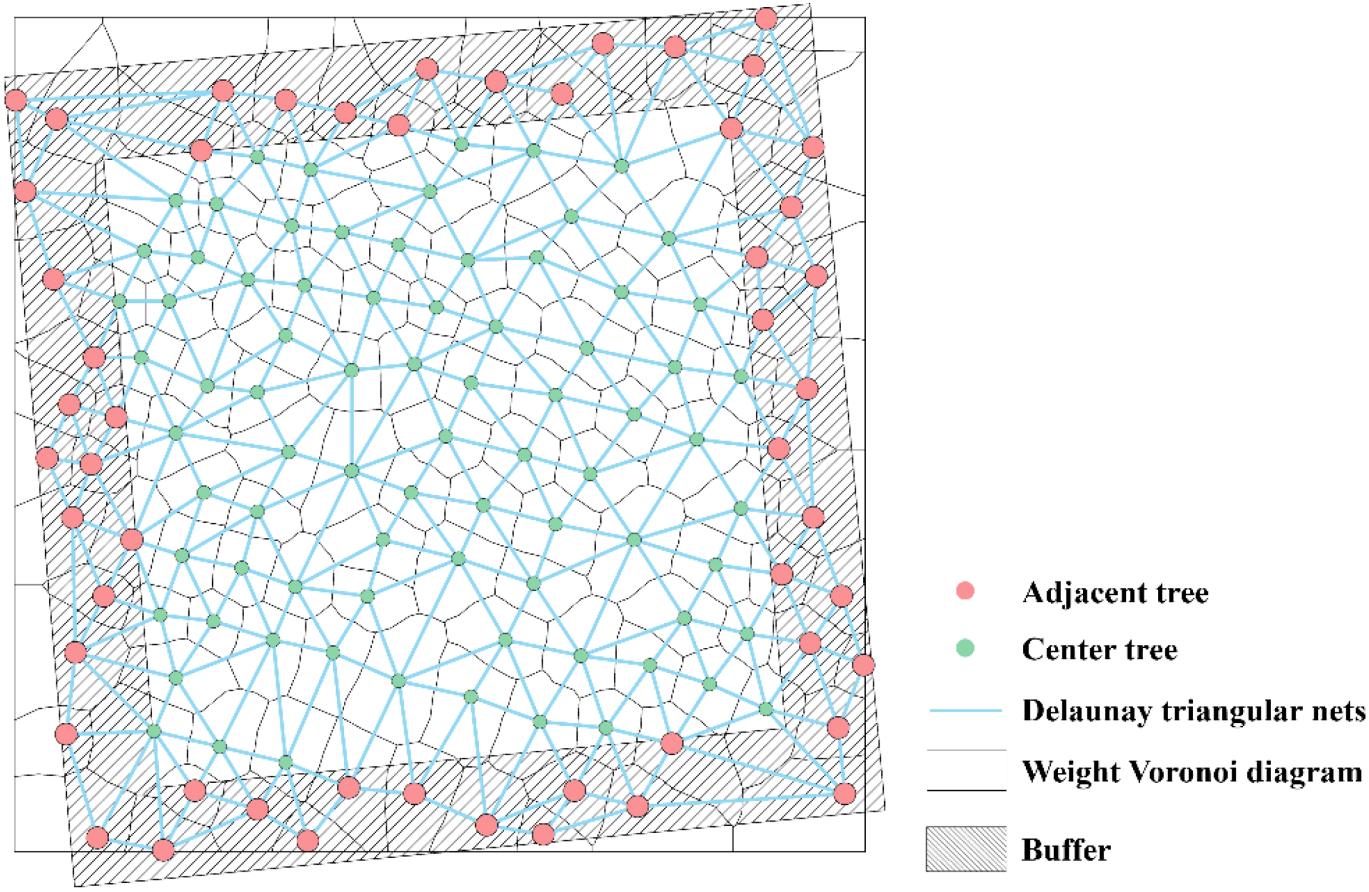
Determination of the spatial structural units of the stand.

Forest tree characteristics, including tree height, CW, and DBH, are essential parameters for assessing tree development status and spatial resource utilization. based on the research of Sun et al. (2022), a weighted Voronoi diagram was constructed using UAV-extracted tree height, DBH, and CW as weight factors to define spatial structural units. A Delaunay triangulation network was then constructed to calculate the distances and angles between target trees and their neighboring trees. To eliminate the edge effect, the distance buffering method (Zhou et al., 2009) was applied, extending the plot to 20 × 20 m. Trees within the buffer zone were included only as neighboring trees (**Figure 4**).

Based on the relationship between the target trees and adjacent trees, the spatial structure parameters of the stand reflect the structural state of the whole stand. Therefore, six spatial structure indices were selected, which were size ratio U (Wan et al., 2020), angle competition index UCI (Hui et al., 2013), openness K (Wang et al., 2013), openness ratio OP (Lin et al., 2021), and uniform angle W (Zhao et al., 2016) and forest layer index S (Cao et al., 2020b). This reflected the spatial distribution of trees in terms of size differentiation, degree of competition, size of growth space, light reception of trees, horizontal distribution, and vertical structure complexity, respectively. The calculation method and meaning are shown in **Table 5**.

**Table 5:** Calculation method of stand spatial structure parameters.

Indices	Formula	Value				
		0	(0,0.25]	(0.25,0.5]	(0.5,0.75]	(0.75,1]
U	$U_{i}=\frac{1}{n}\sum_{j=1}^{n}k_{ij}$ $$t_{ij}=\{\begin{array}{c} 1\;if\;H_{i}<\;H_{j}\\ 0\;if\;H_{i}>\;H_{j} \end{array}$$	superior	sub-superior	moderation	disadvantage	absolute disadvantage
UCI	$UCI_{i}=\frac{U_{i}}{180\times n}\sum_{j=1}^{n}\left(\alpha_{1}+\alpha_{2}\right)$ $$\alpha_{1}=\{\begin{array}{c} \arctan\left(\frac{H_{i}}{d_{ij}}\right)\times \frac{180}{\pi }\;When\;H_{j}>H_{i};\;\\ \arctan\left(\frac{H_{j}}{d_{ij}}\right)\times \frac{180}{\pi }\;\;\;\;\;Otherwise.\;\;\;\;\;\; \end{array}$$ $$\alpha_{2}=\{\begin{array}{c} \arctan\left(\frac{H_{i}-H_{j}}{d_{ij}}\right)\times \frac{180}{\pi }\;When\;H_{j}>H_{i};\\ \;\;\;\;\;\;\;\;0\;\;\;\;\;\;\;\;\;\;\;\;\;\;\;\;\;\;\;\;\;\;\;\;\;\;\;\;Otherwise.\; \end{array}$$	no pressure	less pressure	medium pressure	greater pressure	great pressure
OP	$OP_{i}=\frac{1}{n}\sum_{j=1}^{n}t_{ij}$ $$t_{ij}=\{\begin{array}{c} 1\;if\;d_{ij}>\left|H_{i}-H_{j}\right|\\ 0\;if\;d_{ij}\leq \left|H_{i}-H_{j}\right| \end{array}$$	completely occluded	occluded	medium open	open	extremely open
W	$W_{i}=\frac{1}{n}\sum_{j=1}^{n}t_{ij}$ $$t_{ij}=\{\begin{array}{c} 1\;a_{ij}<a_{0}\\ 0\;a_{ij}\geq a_{0} \end{array};\;a_{0}=\frac{360}{n+1}$$	absolute uniform	uniform	random	aggregation	cluster distributions
S	$S_{i}=\frac{Z_{i}}{3}\times \frac{1}{n}\sum_{j=1}^{n}t_{ij}$ $$t_{ij}=\{\begin{array}{c} 1\;\;When\;i\;and\;j\;belong\;to\;the\;same\;forest\;layer\\ 0\;\;\;\;\;\;\;\;\;\;\;\;\;\;\;\;\;\;\;\;\;\;\;\;\;\;\;Otherwise.\;\;\;\;\;\;\;\;\;\;\;\;\;\;\;\;\;\;\;\;\;\;\;\;\;\;\;\;\;\;\;\;\;\;\;\;\;\; \end{array}$$	single	slightly simple	medium	slightly complex	complex
K	$K_{i}=\frac{1}{n}\sum_{j=1}^{n}\frac{d_{ij}}{H_{ij}}$	Value				
		(0,0.2]	(0.2,0.3]	(0.3,0.4]	(0.4,0.5]	(0.5, +∞)
		serious insufficiency	In-sufficiency	basic sufficiency	sufficient	more than sufficient

*i* represents the central tree and *j* represents the neighboring tree; $H_{i}$ is the height of the tree *i*, $H_{j}$ is the height of the tree *j*, $d_{ij}$ is the distance between the central tree *i* and the neighboring tree *j*, $t_{ij}$ is a discrete variable, $a_{ij}$ represents the minimum angle between *i* and *j*, $a_{0}$ represents the standard angle, *n* represents the number of neighboring trees in the center tree *i*, and $z_{i}$ represents the number of forest layers in the structural unit where the center tree *i* is located. According to Zhou et al. (2019), the height difference (*H_dist_*) between the tallest tree (*H_max_*) and the lowest tree (*H_min_*) in the sample plot was calculated, and then the forest layer was divided, the upper layer: *H*≥*H_min_* +2/3*H_dist_*, the middle layer: *H_min_* +1/3*H_dist_* <*H*<*H_min_* +2/3*H_dist_*, and the lower layer: *H*≤*H_min_* +1/3*H_dist_*.

### 2.5 Statistical analysis

The accuracy of this study’s models was evaluated using R^2^, Root Mean Square Error (RMSE), and Mean Absolute Error Loss (MAE) (Bhatt and Chouhan, 2024). A paired t-test was conducted to compare UAV-extracted tree height and crown width with predicted values. Differences in spatial structure parameters across age groups were analyzed using ANOVA and descriptive statistical analysis. The distribution percentage of biomass across different components within the total aboveground mass was calculated. Path analysis, performed using multiple stepwise regression, examined the impact of stand spatial structure on the proportion of biomass of each component of Chinese fir. Statistical analysis and data modeling were conducted using SPSS Statistics 26, Origin 2022, and Python 3.9.

## 3 Results

### 3.1 Construction of biomass models

According to the results shown in **Table 6**. The formula for calculating the biomass of the aboveground organs of Chinese fir is as follows:

**Table 6:** The results of the construction of the aboveground organ biomass model of Chinese fir.

Organ	Model NO.	Model parameters			R^2^	RMSE	MAE
		a	b	c			
Stem biomass/kg	1	0.022	2.775	–	0.867	19.132	16.221
	2	0.108	2.083	–	0.977	7.948	7.026
	3	0.054	0.786	–	0.980	7.361	6.054
	4	14.623	0.006	–	0.979	7.691	6.457
	5	-2.570	2.190	–	0.960	0.157	0.131
	6	0.297	0.252	-0.003	0.956	0.165	0.142
	7	-2.387	2.291	–	0.900	0.248	0.218
	8	-2.755	0.768	–	0.976	0.123	0.099
	9	-2.753	1.552	0.752	0.976	0.122	0.097
Branch biomass/kg	1	0.001	3.103	–	0.826	3.947	2.934
	2	0.007	2.353	–	0.946	2.194	1.464
	3	0.003	0.888	–	0.946	2.190	1.338
	4	1.007	0.001	–	0.944	2.244	1.433
	5	-5.718	2.582	–	0.962	0.180	0.154
	6	-2.374	0.301	-0.004	0.961	0.181	0.153
	7	-5.454	2.685	–	0.890	0.306	0.261
	8	-5.920	0.904	–	0.973	0.150	0.115
	9	-5.907	1.921	0.779	0.974	0.150	0.118
Leaf biomass/kg	1	0.937	0.828	–	0.871	0.939	0.708
	2	1.291	0.666	–	0.830	1.077	0.928
	3	1.106	0.245	–	0.869	0.943	0.789
	4	7.395	0.001	–	0.648	1.469	1.277
	5	-0.078	0.771	–	0.840	0.117	0.104
	6	0.327	0.148	-0.002	0.902	0.092	0.073
	7	-0.187	0.868	–	0.912	0.088	0.069
	8	-0.200	0.277	–	0.894	0.096	0.083
	9	-0.238	0.214	0.656	0.922	0.081	0.066

Stem biomass model (Equation 4):


(4) $$W_{stem}=0.054{\left(D^{2}H\right)}^{0.786}$$

Branch biomass model (Equation 5):


(5) $$lnW_{branch}=-5.907+1.921lnD+0.779lnH$$

Leaf biomass model (Equation 6):


(6) $$lnW_{leaf}=-0.238+0.214lnD+0.656lnH$$

where *W_stem_* represents stem biomass, *W_branch_* represents branch biomass, *W_leaf_* represents leaf biomass, *D* represents tree diameter at breast height, and *H* represents tree height.

### 3.2 Calculation of stand spatial structure parameters

#### 3.2.1 Extraction of forest parameters

The results of single-tree segmentation are presented in **Table 7**, indicating varying optimal window combinations across different plots. Tree vertex detection accuracy ranged from 0.809 to 0.943. The detection accuracies for young, middle-aged, near-ripe, mature, and overripe forests were 0.893, 0.856, 0.843, 0.879, and 0.849, respectively. The local maximum algorithm achieved detection precision exceeding 0.8 in all sample plots. Across all plots, the mean accuracy was 0.859, indicating strong performance in tree vertex extraction.

**Table 7:** Optimal window combination for each plot.

Sample plot NO.	Age Group	Window combination	Observed trees	Detecting trees	Correct amount	Number of errors	Number of missed points	R	P	F
Y1	middle-aged forest	0.2×0.2	187	177	158	19	29	0.845	0.893	0.868
Y2	middle-aged forest	0.1×0.1	250	189	178	11	72	0.712	0.942	0.811
Y3	mature forest	0.2×0.3	152	120	112	8	40	0.737	0.933	0.824
Y4	overripe forest	0.3×0.3	113	116	108	8	5	0.956	0.931	0.943
Y5	overripe forest	0.3×0.3	92	100	87	13	5	0.946	0.87	0.906
Y6	near-ripe	0.2×0.2	160	153	142	11	18	0.888	0.928	0.907
Y7	overripe forest	0.3×0.2	112	126	98	28	14	0.875	0.778	0.824
Y8	near-ripe	0.3×0.2	157	139	125	14	32	0.796	0.899	0.845
Y9	near-ripe	0.2×0.1	149	155	129	26	20	0.866	0.832	0.849
Y10	middle-aged forest	0.4×0.4	83	70	69	1	14	0.831	0.986	0.902
Y11	middle-aged forest	0.4×0.4	91	89	83	6	8	0.912	0.933	0.922
Y12	middle-aged forest	0.3×0.4	74	71	59	12	15	0.797	0.831	0.814
Y13	overripe forest	0.4×0.4	50	53	44	9	6	0.880	0.830	0.854
Y14	overripe forest	0.4×0.5	44	48	40	8	4	0.909	0.833	0.870
Y15	mature forest	0.5×0.6	40	41	34	7	6	0.85	0.829	0.840
Y16	overripe forest	0.3×0.2	102	108	85	23	17	0.833	0.787	0.810
Y17	overripe forest	0.2×0.3	114	116	101	15	13	0.886	0.871	0.878
Y18	overripe forest	0.1×0.2	125	116	99	17	26	0.792	0.853	0.822
Y19	overripe forest	0.1×0.2	118	115	103	12	15	0.873	0.896	0.884
Y20	mature forest	0.1×0.2	139	122	112	10	27	0.806	0.918	0.858
Y21	near-ripe	0.2×0.2	135	106	99	7	36	0.733	0.934	0.822
Y22	near-ripe	0.2×0.2	152	121	111	10	41	0.730	0.917	0.813
Y23	middle-aged forest	0.2×0.2	156	133	119	14	37	0.763	0.895	0.824
Y24	near-ripe	0.2×0.3	118	108	97	11	21	0.822	0.898	0.858
Y25	mature forest	0.5×0.5	62	71	56	15	6	0.903	0.789	0.842
Y26	mature forest	0.5×0.3	88	90	72	18	16	0.818	0.800	0.809
Y27	mature forest	0.2×0.5	75	77	64	13	11	0.853	0.831	0.842
Y28	overripe forest	0.5×0.4	47	42	39	3	8	0.830	0.929	0.876
Y29	overripe forest	0.4×0.4	79	71	62	9	17	0.785	0.873	0.827
Y30	overripe forest	0.5×0.5	40	43	38	5	2	0.950	0.884	0.916
Y31	young forest	0.2×0.5	152	132	130	2	22	0.855	0.985	0.915
Y32	young forest	0.1×0.4	162	152	143	9	19	0.883	0.941	0.911
Y33	young forest	0.1×0.2	193	170	155	15	38	0.803	0.912	0.854

**Figure 5:**
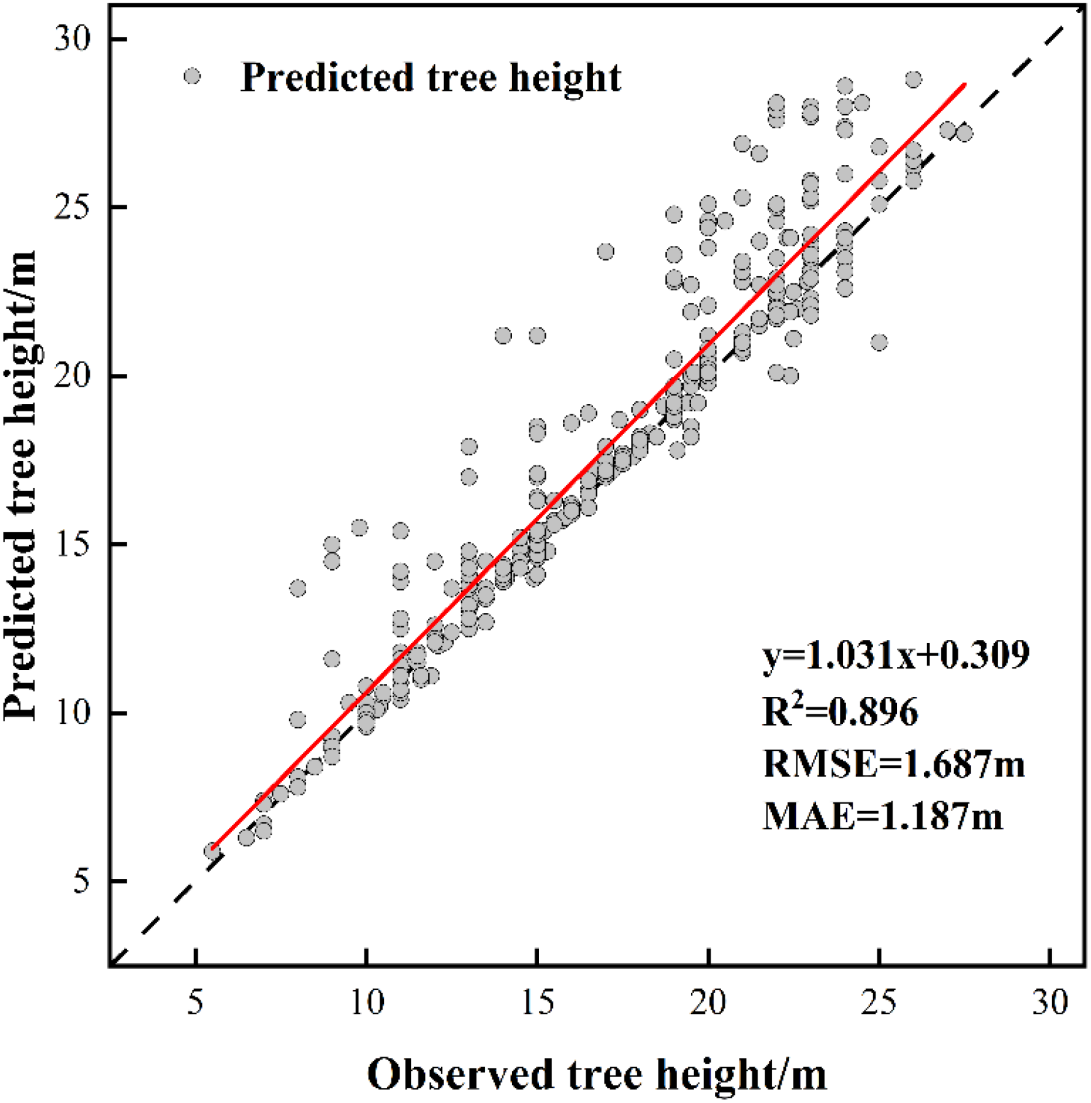
The fitting result of predicted tree height and observed tree height.

**Figure 6:**
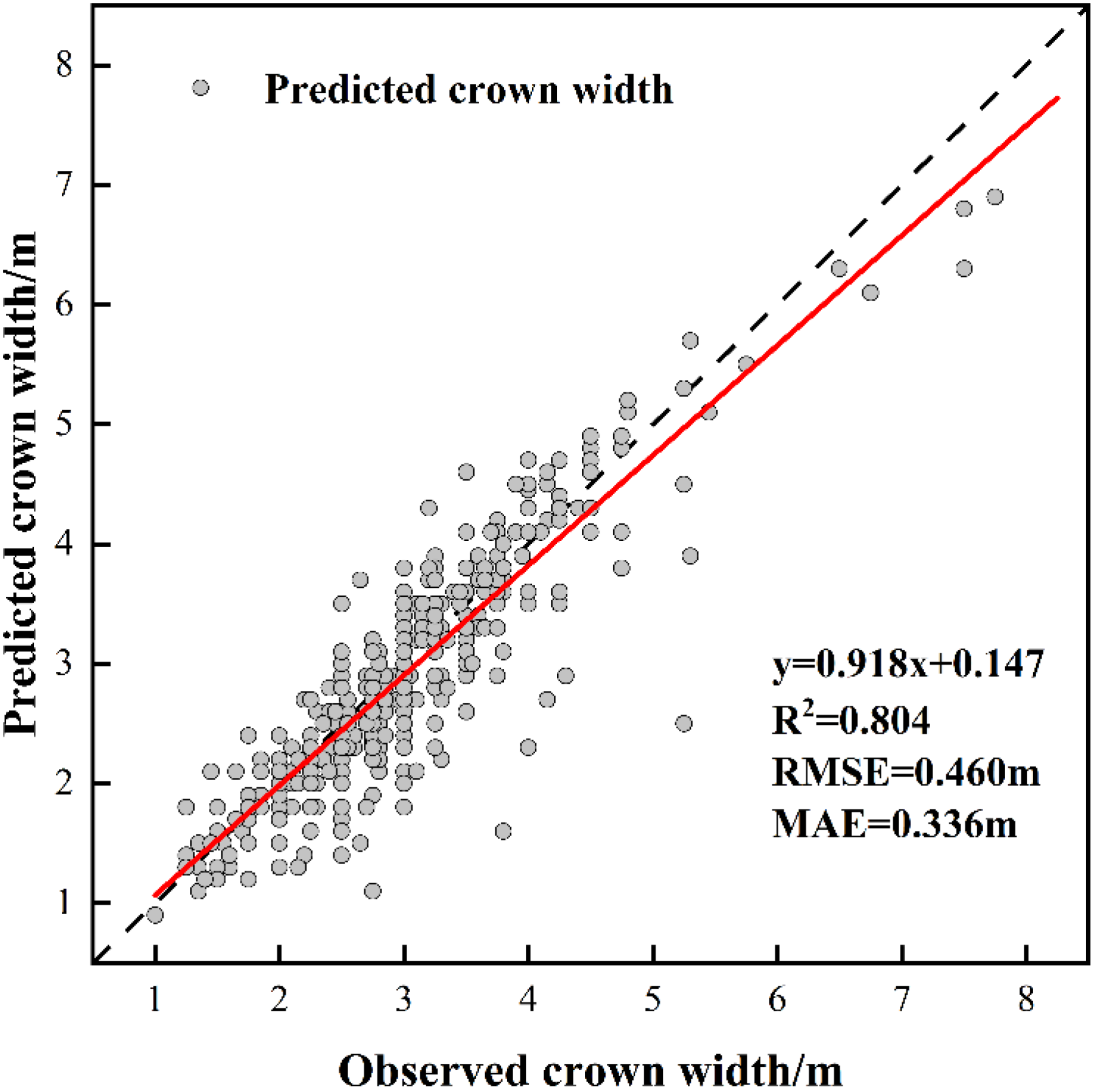
The fitting result of predicted CW and observed CW.

The measurements of tree height and crown width were obtained from individual sample plots, and validation was conducted by comparing UAV-extracted data against ground-truth measurements from 353 trees with RTK positioning data (**Table 8**). The validation outcomes for tree height are displayed in **Figure 5**, crown width validation results appear in **Figure 6** and **Table 9**. These results demonstrate no statistically significant variation between field-measured and UAV- predicted values for both parameters, confirming the high precision of UAV-based estimation methods for Chinese fir dimensions.

**Table 8:** The paired t-test of predicted tree height and observed tree height.

Difference			T	df	p
Mean value	SD	SE			
0.109	1.297	0.105	1.041	352	0.299

p-value indicates significance, p>0.05 means no significant difference, p<0.05 means significant difference, SD means Standard deviation, SE means Standard error.

**Table 9:** The paired t-test of predicted CW and observed CW.

Difference			T	df	p
Mean value	SD	SE			
0.039	0.357	0.029	1.357	352	0.177

p-value indicates significance, p>0.05 means no significant difference, p<0.05 means significant difference, SD means Standard deviation, SE means Standard error.

#### 3.2.2 DBH model construction results

According to the Pearson correlation test, there is a strong positive correlation between UAV-derived predictions of tree height(r=0.771, p<0.05) and CW (r=0.589, p<0.05)with measured DBH values. Based on this relationship, multiple linear regression analysis was employed to develop a DBH model.

**Figure 7:**
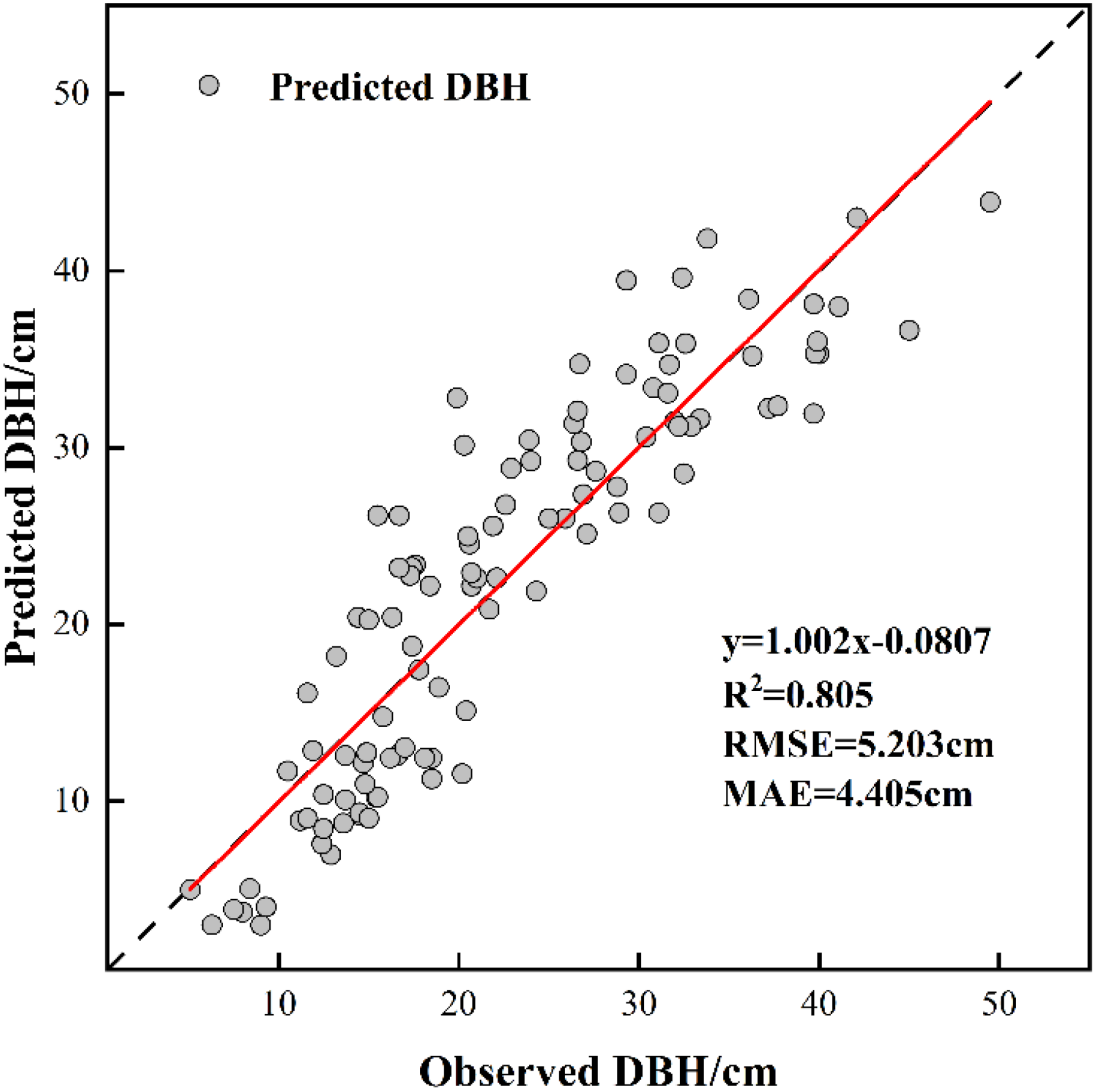
DBH model validation results.


The training outcomes of the developed model are presented in **Figure 7**. With a determination coefficient of R^2^ = 0.673 and absence of multicollinearity issues, the established model demonstrates statistical validity(VIF<5). According to the results of the paired t-test(T=1.097, p=0.142>0.1), there was no significant difference between the observed and predicted DBH, the predictive results of the constructed DBH model are better and can be used for the inversion of DBH of Chinese fir. The formula used is as follows (Equation 7):

(7) DBH=1.475×H+2.965×CW−13.117

#### 3.2.3 Analysis of the spatial structure characteristics of Chinese fir plantations

**Table 10** shows the variation in spatial structure parameters across different Chinese fir age stages. The angular scale reflects the horizontal spatial distribution of trees, and results indicate a significant difference across growth stages (P<0.01). The average angular scale of young and middle-aged Chinese fir forests was significantly higher than that of other stages. The average uniform angle for young, middle-aged, near-mature, and mature forests was 0.625, 0.553, 0.526, and 0.501, respectively, all indicating an aggregated distribution pattern. However, as Chinese fir aged, the horizontal distribution structure gradually evolved to a random distribution, becoming completely random in overripe forests, where the average uniform angle reached 0.496.

**Table 10:** Spatial structure parameters of Chinese fir plantations at different ages.

Age group	Spatial structure parameters					
	W	U	UCI	S	K	OP
Young forest	0.625 ± 0.108^A^	0.514 ± 0.014	0.267 ± 0.011	0.353 ± 0.074^b^	0.246 ± 0.016^A^	0.916 ± 0.025^A^
Middle-aged forest	0.553 ± 0.041^A^	0.548 ± 0.037	0.293 ± 0.033	0.415 ± 0.076^ab^	0.240 ± 0.024^A^	0.880 ± 0.041^A^
Near-ripe forest	0.526 ± 0.028^B^	0.522 ± 0.029	0.298 ± 0.014	0.424 ± 0.085^ab^	0.187 ± 0.047^B^	0.804 ± 0.059^B^
Mature forest	0.501 ± 0.016^C^	0.512 ± 0.032	0.288 ± 0.021	0.443 ± 0.053^ab^	0.188 ± 0.033^B^	0.796 ± 0.092^B^
Overripe forest	0.496 ± 0.025^C^	0.500 ± 0.019	0.283 ± 0.024	0.513 ± 0.104^a^	0.164 ± 0.027^C^	0.784 ± 0.226^C^

The uppercase letters in the table indicate a very significant difference, p<0.05, and the lowercase letters in the table indicate a significant difference, p<0.1.

The size ratio reflected the growth differences among trees within a structural unit. There were either no significant differences or small differences among the five age stages (P > 0.05), with values of 0.514, 0.548, 0.522, 0.512, and 0.500, respectively. However, the overall trend suggests that the size ratio of Chinese fir forests gradually decreases with age, indicating a shift from inferior to moderate growth.

The competition index of the intersection angle reflects the degree of competition for survival resources among trees. No significant differences were observed across the five age stages (P>0.05). The average competition index for young, middle-aged, near-mature, mature, and overripe forests was 0.267, 0.293, 0.298, 0.288, and 0.283, respectively, all reflecting moderate competitive pressure.

The forest layer index represents vertical space utilization and structural complexity. The average layer indices for Chinese fir plantations at the five age stages were 0.353, 0.415, 0.424, 0.443, and 0.513, respectively, with significant differences observed (P<0.05). The layer index was notably higher in overripe plantations than in the other four stages. The general trend indicated that as Chinese fir ages, the forest layer index increased, with the vertical structure evolving from simple to complex.

Openness and openness ratios reflect understory and canopy light exposure. Significant differences were found across age groups (P< 0.01). The openness values for the five age stages were 0.246, 0.240, 0.187, 0.188, and 0.164, respectively, showing a continuous decline. This suggests that as Chinese fir grows, light transmission in the understory decreases due to increasing canopy cover, restricting growth space. The openness ratios by age group were 0.916, 0.880, 0.804, 0.796, and 0.784, also showing a decreasing trend. The combined changes in these spatial structure indices indicate that as Chinese fir matures, canopy coverage increases, leading to reduced light penetration within the forest.

### 3.3 Effects of stand spatial structure on AGB distribution of Chinese fir

#### 3.3.1 Screening of key spatial structure factors based on path analysis

The effect of stand spatial structure on the biomass mass fraction of each Chinese fir component was analyzed using path analysis. The size ratio was excluded from the regression model due to collinearity with the angle competition index.

**Figure 8:**
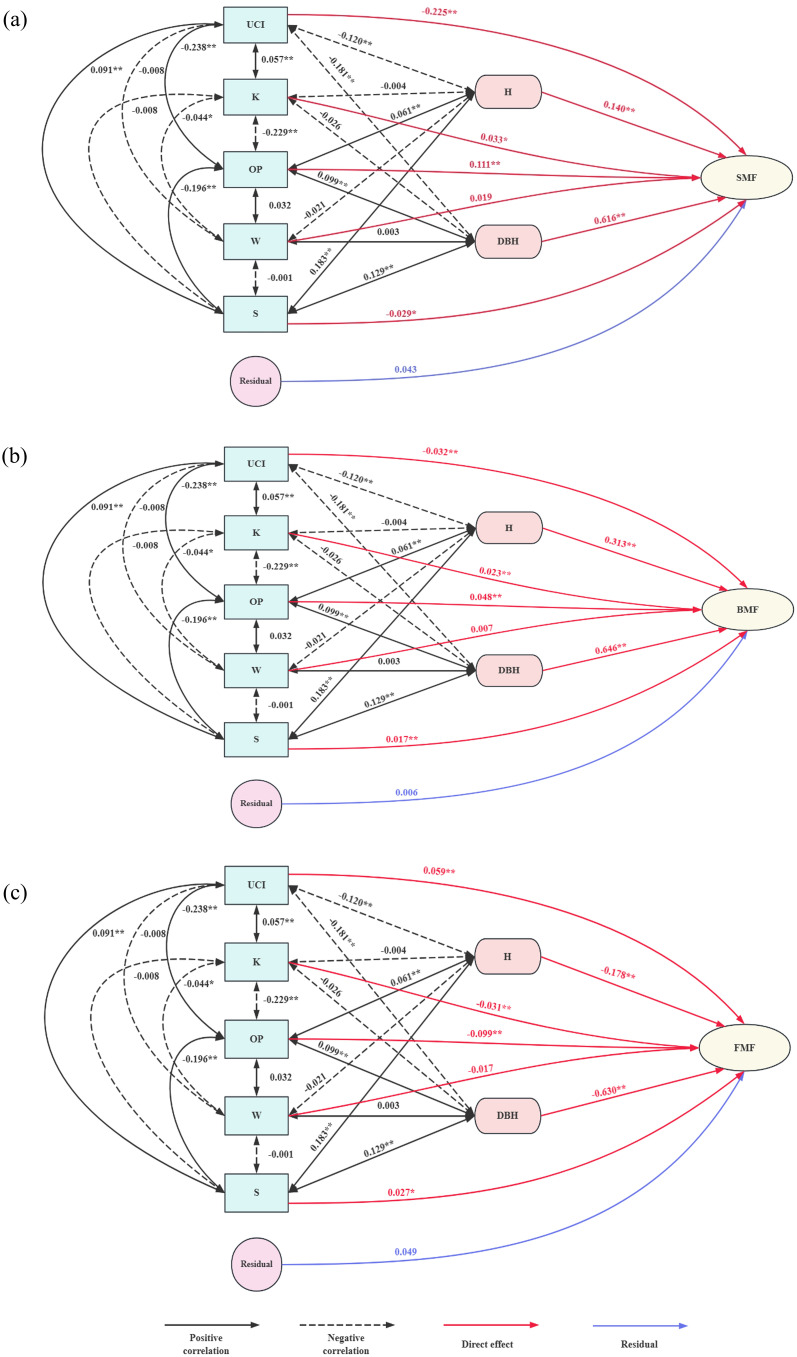
Path analysis of the influence of independent variables on dependent variables. **(a)** Path analysis diagram of stand structure factors and SMF; **(b)** Path analysis diagram of stand structure factors and BMF; **(c)** Path analysis diagram of stand structure factors and FMF.

The path analysis results are presented in **Table 11** and **Figure 8**(Please find attached for more details). DBH was the most directly affected variable, followed by tree height. The influence of spatial structure factors was primarily exerted through DBH and tree height, either enhancing or restricting the biomass proportion of each component. The uniform angle had a minimal effect on biomass mass fractions, and its effect was not significant.

**Table 11:** Path analysis between the spatial structure of the stand and the biomass fraction.

Organ	Factor	Correlation coefficient	Direct diameter factor	Indirect diameter factor								Decision coefficient	Residual
				UCI	K	OP	W	S	H	DBH	Total		
SMF	UCI	-0.225^**^	-0.065^**^	–	0.002	-0.026	0.000	-0.003	-0.017	-0.112	-0.160^**^	0.025	0.043
	K	-0.014	0.033^*^	-0.004	–	-0.025	-0.001	0.000	-0.001	-0.016	-0.047	0.002	
	OP	0.195^**^	0.111^**^	0.015	-0.008	–	0.001	0.006	0.009	0.061	0.084^*^	0.031	
	W	0.021	0.019	0.001	-0.001	0.004	–	0.000	-0.003	0.002	0.002	0.000	
	S	0.048^*^	-0.029^*^	-0.006	0.000	-0.022	0.000	–	0.026	0.079	0.077^*^	0.004	
	H	0.722^**^	0.140^**^	0.008	0.000	0.007	0.000	-0.005	–	0.573	0.582^**^	0.182	
	DBH	0.765^**^	0.616^**^	0.012	-0.001	0.011	0.000	-0.004	0.130	–	0.149^**^	0.563	
BMF	UCI	-0.197^**^	-0.032^**^	–	0.001	-0.011	0.000	0.002	-0.038	-0.117	-0.165^**^	0.012	0.006
	K	-0.009	0.023^**^	-0.002	–	-0.011	0.000	0.000	-0.001	-0.017	-0.032^*^	0.001	
	OP	0.130^**^	0.048^**^	0.008	-0.005	–	0.000	-0.003	0.019	0.064	0.082^*^	0.011	
	W	0.004	0.007	0.000	-0.001	0.001	–	0.000	-0.006	0.002	-0.004	0.000	
	S	0.146^**^	0.017^**^	-0.003	0.000	-0.009	0.000	–	0.057	0.083	0.128^**^	0.004	
	H	0.924^**^	0.313^**^	0.004	0.000	0.003	0.000	0.003	–	0.601	0.611^**^	0.477	
	DBH	0.950^**^	0.646^**^	0.006	-0.001	0.005	0.000	0.002	0.291	–	0.304^**^	0.804	
FMF	UCI	0.223^**^	0.059^**^	–	-0.002	0.024	0.000	0.002	0.021	0.114	0.164^**^	0.023	0.049
	K	0.013	-0.031^**^	0.003	–	0.023	0.001	0.000	0.001	0.017	0.044	0.002	
	OP	-0.185^**^	-0.099^**^	-0.014	0.007	–	-0.001	-0.005	-0.011	-0.062	-0.086^*^	0.027	
	W	-0.018	-0.017	0.000	0.001	-0.003	–	0.000	0.004	-0.002	-0.001	0.000	
	S	-0.062^**^	0.027^*^	0.005	0.000	0.019	0.000	–	-0.033	-0.081	-0.089^*^	0.004	
	H	-0.772^**^	-0.178^**^	-0.007	0.000	-0.006	0.000	0.005	–	-0.586	-0.595^**^	0.243	
	DBH	-0.813^**^	-0.630^**^	-0.011	0.001	-0.010	0.000	0.003	-0.165	–	-0.182^**^	0.628	

In the table, ** represents p<0.05, i.e., there is a very significant correlation, and * indicates p<0.1, i.e., there is a significant correlation.

For SMF, the openness ratio and openness had significant positive direct effects, while the angle competition index and forest layer index had notable negative direct effects. The openness ratio also had a strong positive indirect effect on SMF through other factors, resulting in a significant overall correlation. Conversely, the competition index had a significant negative indirect effect on SMF, leading to a strong negative correlation. Although openness had a significant positive direct effect on SMF, its indirect limiting effects outweighed its direct positive effect, making the correlation with SMF negative and not significant. The forest layer index had a negative direct effect but a significant positive correlation with SMF by influencing DBH and tree height. Based on decision coefficients, the order from largest to smallest is the openness ratio, angle competition index, forest layer index, and openness.

For BMF, the openness ratio, openness, and forest layer index had significant positive direct effects, while the competition index had a notable negative direct effect. The openness ratio and forest layer index also had significant positive indirect effects on BMF by influencing tree height, DBH, and other spatial structure factors, resulting in a strong positive correlation with BMF. The competition index limited BMF through its effects on tree height and DBH, leading to a significant negative indirect effect. The negative indirect effect of openness on BMF was greater than its positive direct effect, making its overall correlation with BMF not significant. Based on decision coefficients, the order from largest to smallest is the competition index, openness ratio, forest layer index, and openness.

For FMF, the openness ratio and openness exhibited a strong negative direct effect, while the competition and forest layer indices had significant positive direct effects. The openness ratio also had a significant negative indirect effect on FMF, leading to a strong negative correlation. Although openness had both positive and indirect effects, they were weaker than its negative direct effect, resulting in no significant positive correlation with FMF. The competition index, aside from its negative indirect effect through openness, had positive indirect effects through other indicators, leading to a highly significant positive correlation with FMF. The forest layer index had a significant negative indirect effect on FMF through tree height and DBH, which outweighed its positive direct effect, resulting in a strong negative correlation. Based on decision coefficients, the order from largest to smallest is the openness ratio, competition index, forest layer index, and openness.

#### 3.3.2 Four-element distribution of SMF, BMF, and FMF

After identifying OP, UCI, S, and K as the main spatial structure factors influencing AGB allocation in Chinese fir, a four-element distribution map was constructed to analyze biomass allocation under different spatial structure combinations. Since each spatial structure factor had five values, UCI&K were used as the x-axis and OP&S as the y-axis, resulting in 625 spatial structure combinations.

**Figure 9:**
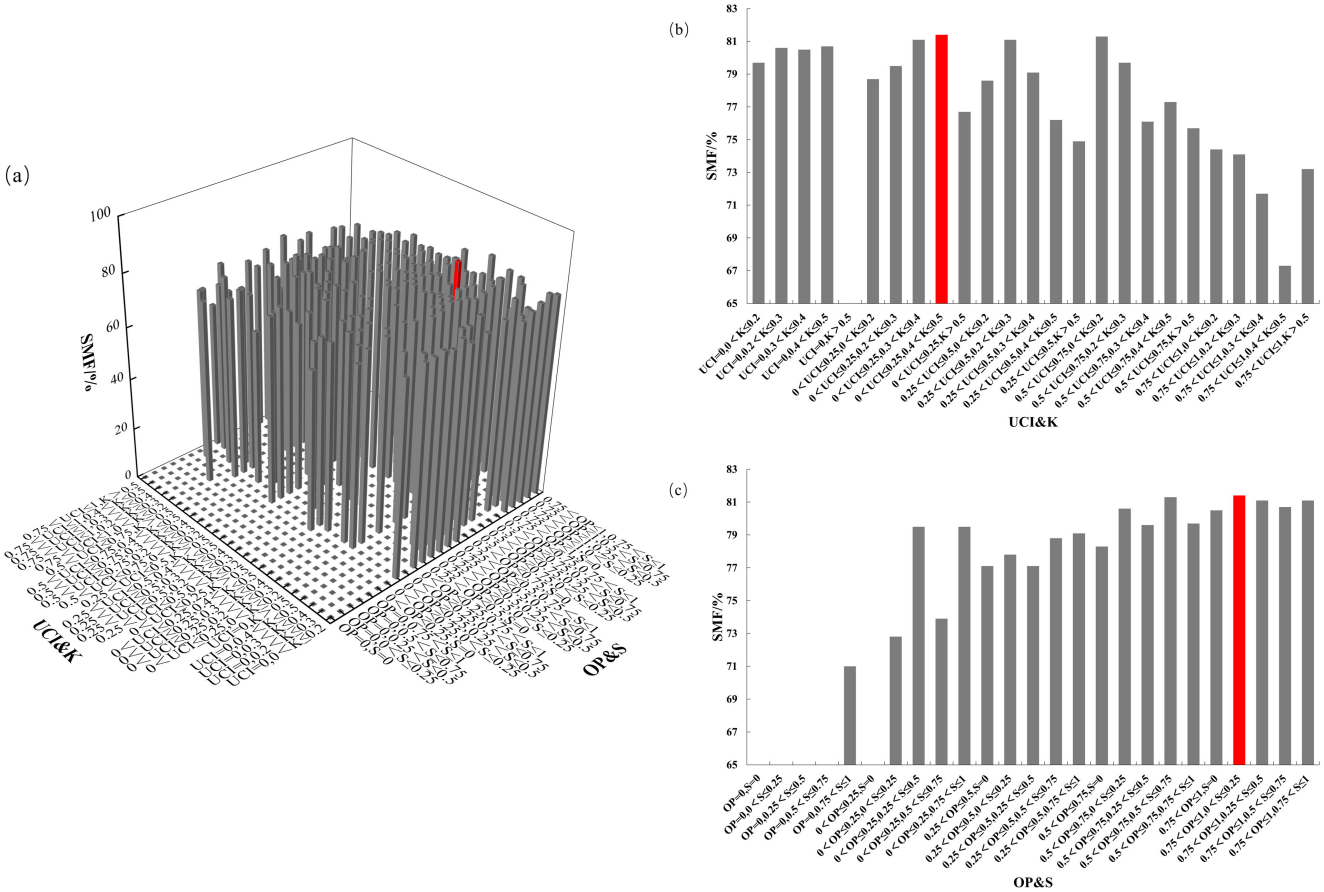
SMF distribution under different spatial structure combinations. **(a)** the four-element distribution of SMF; **(b)** the four-element distribution from the perspective of UCI&K; **(c)** the four-element distribution from the perspective of OP&S.

The four-element distribution of SMF is shown in **Figure 9**. The SMF ranged from 0% to 81.4%, with almost no trees having OP = 0. From the perspective of UCI&K, SMF decreased as the competition index increased, reaching its highest value when 0< UCI ≤ 0.25 and 0.4< K ≤ 0.5. From the OP&S perspective, SMF increased with a higher openness ratio, peaking at 0.75< OP ≤ 1 and 0< S ≤ 0.25. The mass fraction of Chinese fir trunk biomass was maximized under conditions of low competition pressure, ample living space, high light availability, and a relatively simple forest layer structure.

**Figure 10:**
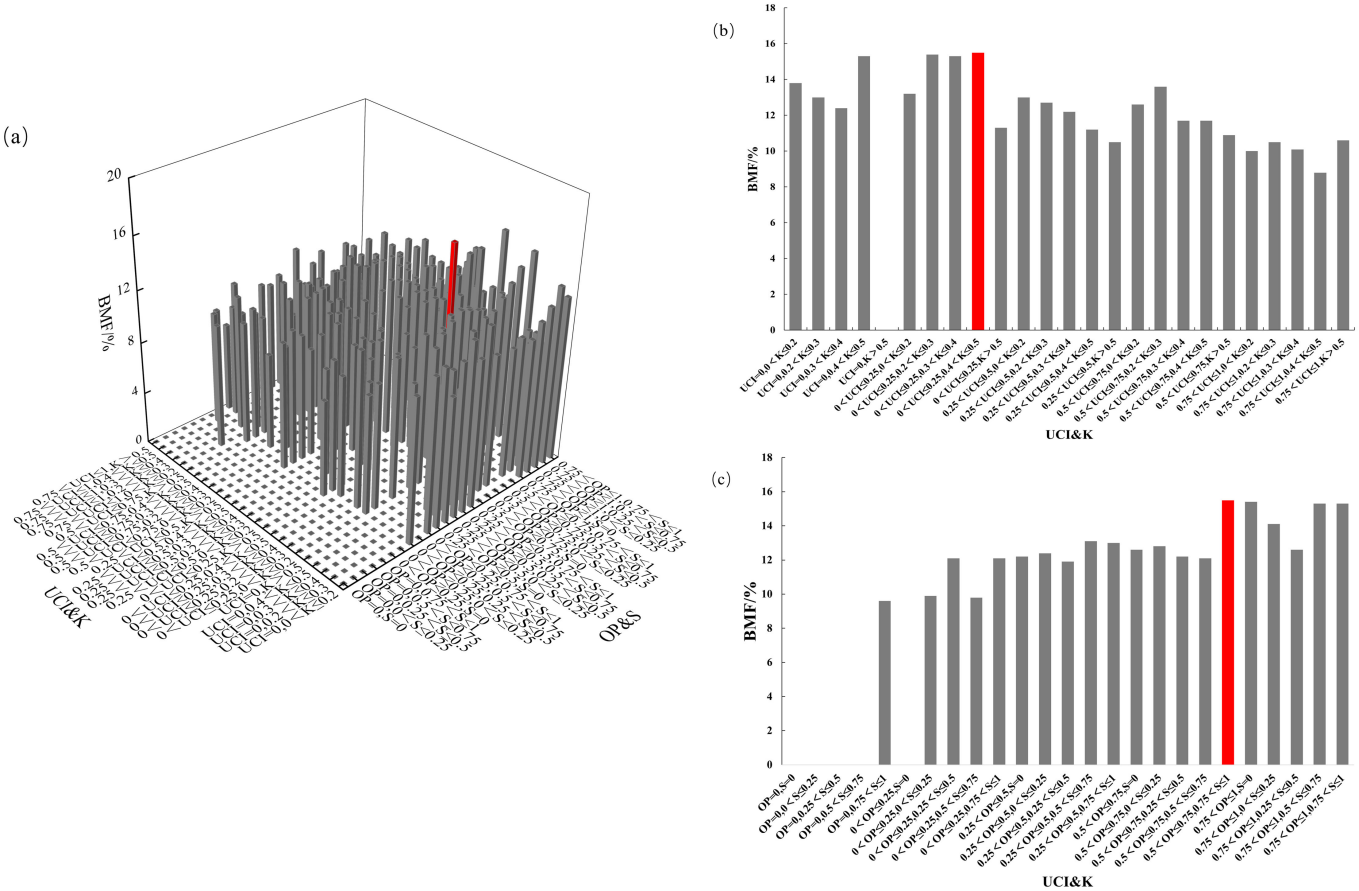
BMF distribution under different spatial structure combinations. **(a)** the four-element distribution of BMF; **(b)** the four-element distribution from the perspective of UCI&K; **(c)** the four-element distribution from the perspective of OP&S.

The four-element distribution of BMF in Chinese fir is shown in **Figure 10**. The value of BMF ranged from 0% to 15.5%. From the perspective of UCI&K, the BMF displayed a diminishing trend with the increase of the competition index, in which the BMF was the highest when the combination of 0<UCI ≤ 0.25, 0.4<K ≤ 0.5. From the perspective of OP&S, the BMF showed a trend of increasing with the increase of the open ratio. The BMF was the highest when the combination of 0.5<OP ≤ 0.75, 0.75<S ≤ 1. The mass fraction of Chinese fir branch biomass reaches its maximum when competition pressure is low, the living environment is sufficient, light availability is high, and the forest layer structure is complex.

**Figure 11:**
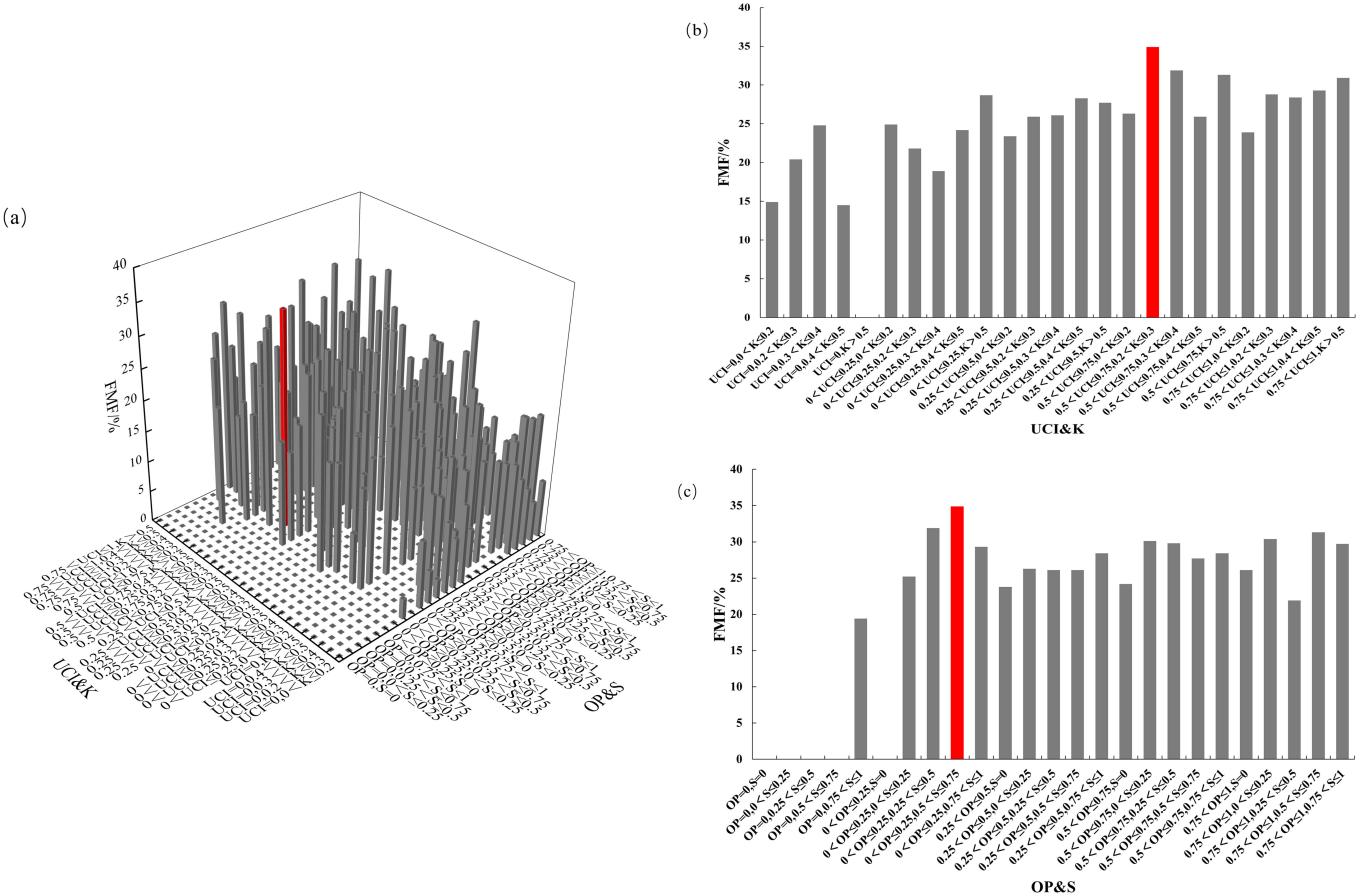
FMF distribution under different spatial structure combinations. **(a)** the four-element distribution of FMF; **(b)** the four-element distribution from the perspective of UCI&K; **(c)** the four-element distribution from the perspective of OP&S.

The four-element distribution of the Chinese fir FMF is shown in **Figure 11**. The value range of the FMF was 0–34.9%. From the perspective of UCI & K, FMF showed an increasing trend when the UCI of forest trees gradually increased. FMF reached its highest when Chinese fir were in the combination of 0.5< UCI ≤ 0.75 and 0.2< K ≤ 0.3. From the perspective of OP&S, large FMF mainly appears when 0< OP ≤ 0.25; that is, when Chinese fir is in the combination of 0< OP ≤ 0.25 and 0.5< S ≤ 0.75, FMF will reach the highest. The biomass fraction of Chinese fir leaves reached a maximum when the competition pressure of Chinese fir was high, the living environment was insufficient, the light was blocked, and the forest layer structure was more complex.

OP, UCI, S, and K were the four major spatial structural factors affecting the aboveground biomass distribution pattern of Chinese fir. When the four spatial structure factors were combined, as shown in **Table 12**, the biomass mass fraction of the corresponding components of Chinese fir reached a maximum. Therefore, the aboveground biomass distribution pattern of Chinese fir could be altered by adjusting the spatial structure of stands.

**Table 12:** Effects of OP, UCI, S and K on aboveground biomass allocation pattern of Chinese fir.

Organ	Spatial structure parameters			
	OP	UCI	S	K
SMF	0.75<OP ≤ 1	0<UCI ≤ 0.25	0<S ≤ 0.25	0.4<K ≤ 0.5
BMF	0.5<OP ≤ 0.75	0<UCI ≤ 0.25	0.75<S ≤ 1	0.4<K ≤ 0.5
FMF	0<OP ≤ 0.25	0.5<UCI ≤ 0.75	0.5<S ≤ 0.75	0.2<K ≤ 0.3

## 4 Discussion

### 4.1 UAV parameter extraction

With the application of UAV technology in forestry, airborne LiDAR, with its superior convenience, penetration, and applicability, can describe ground object information with high precision and has unique advantages in reconstructing complex forest vertical structures (Tang et al., 2019). Therefore, it is widely used to determine the forest tree parameters (Almeida et al., 2019; Ferraz et al., 2016).

In this study, the extraction of tree parameters and spatial structure parameters was based on the results of tree top extraction; therefore, the accuracy of tree top extraction is a key factor affecting parameter extraction. Since Chinese fir is a coniferous species with a distinct advantage in tree vertices, a circular window was used to extract tree vertices through a local maximum algorithm. The overall detection accuracy for young forests was 0.893, for middle-aged forests it was 0.856, for near-mature forests it was 0.843, for mature forests it was 0.879, and for over-mature forests it was 0.849. The detection results for young forests were better than those of other age groups, and there was a noticeable trend that tree top detection results decreased as the age of the Chinese fir increased, due to the increasing density of the forest and the overlap of tree crowns leading to blurred edges between trees. Some studies have shown that the mutual occlusion of the canopy can affect the detection results of the vertices of single trees (Brunner and Houtmeyers, 2022). Therefore, it may be possible to further improve the accuracy of Chinese fir tree vertices detection by using a variable maximum detection window and adjusting the CHM pixel size (Brieger et al., 2019; Picos et al., 2020). In this study, using the local maximum value algorithm yielded high accuracy in extracting tree vertices, with an average accuracy of 0.859. The local maximum value algorithm has strong applicability for extracting treetops in coniferous forests (Mohan et al., 2017), aligning with the findings obtained in this investigation.

In terms of tree parameter extraction, the tree height extraction accuracy was R²= 0.896, RMSE = 1.687 m, and MAE = 1.187 m. The crown-width extraction accuracy was R²= 0.804, RMSE = 0.460 m, and MAE = 0.336 m, indicating a high level of precision. This high accuracy was largely due to the small sample plot area, relatively flat terrain, and uniform tree species, which created favorable conditions for extracting tree vertices and parameters (Zhang et al., 2023). However, even under such conditions, errors in forest tree parameter extraction persist. LiDAR is a high-precision three-dimensional geographic data acquisition technology based on laser pulse emission and echo reception. It measures the time difference of light pulse propagation to determine the distance between the target object and the sensor (Cheng et al., 2024). Variations in UAV flight height affect the quality of point cloud data generation. Flying too low causes data redundancy and increases processing difficulty, whereas flying too high may reduce or eliminate ground point clouds, thus failing to generate accurate DEM data (Liu et al., 2022). In order to obtain more accurate parameter extraction results, in the follow-up research, we will explore the influence of different flight altitudes on individual tree segmentation and forest parameters, and also combine airborne lidar and ground lidar to improve the quality of point cloud data, so as to further improve the accuracy of parameter extraction.

In addition to the quality of the point cloud, the accuracy of individual tree segmentation will also have an impact on the extraction of forest parameters. In this study, the watershed algorithm was used to segment the tree canopy. The principle of the watershed algorithm is derived from the concept of watersheds, which can treat each watershed as a closed basin formed by the connection of ridgelines, and apply it to the division of individual trees in the forest, and each watershed corresponds to a canopy (Wang et al., 2010). The processing process of the watershed algorithm is to filter the original lidar data by point cloud filtering, separate ground points and non-ground points, interpolate the corresponding DEM and DSM, and subtract DEM from DSM to obtain the canopy height model (CHM=DSM-DEM). When generating DEM and DSM, it is necessary to rasterize and interpolate the point cloud data, and at the same time, the DSM data needs to be filled with invalid values. Due to the corresponding smoothing and removal of the data in this process, part of the information of the original point cloud data is lost, which affects the accuracy of the later single-wood segmentation to a certain extent, which shows that the watershed single-timber segmentation has a high dependence on the accuracy of DEM and DSM, so we will also explore the influence of different interpolation methods and smoothing methods on the segmentation results in the future.

### 4.2 Effects of spatial structure on biomass allocation of aboveground various organs of Chinese fir

Plants adapt to their environment by regulating biomass allocation among different organs, maximizing resource acquisition (light, water, and nutrients) to maintain optimal growth (Poorter and Nagel, 2000; Xie et al., 2016; Zhou et al., 2023). The findings indicate that spatial structure factors influence biomass proportions primarily through DBH and tree height, either facilitating or restricting resource allocation to different components of Chinese fir. This suggests that stand spatial structure does not independently determine biomass ratios but interacts with the growth characteristics of individual trees. A well-structured stand can enhance resource availability, such as light, water, and nutrients, thereby promoting DBH and tree height growth (Ali, 2019; Zhang et al., 2019) and indirectly affecting biomass proportion. Among the spatial structure indicators, the openness ratio, angle competition index, openness, and forest layer index had the most prominent influence. In plant communities, light conditions and available growth space are key factors affecting tree growth (Chiang et al., 2019; Lundqvist and Elfving, 2010). The competition index, openness, and forest layer index reflect spatial utilization in both horizontal and vertical dimensions, while the openness ratio indicates how effectively trees use light. Together, these indicators illustrate the adaptive strategies of Chinese fir under varying growth conditions. As observed in this study, when light conditions were favorable, competition pressure was low, and survival space was sufficient, Chinese fir allocated more resources to trunk and branch growth. Enhanced photosynthesis increases organic matter production, providing the necessary energy and material for tree development. Since trunks and branches support the tree structure and facilitate nutrient and water transport (Maier, 2001), trees prioritize their growth when resources are abundant. Conversely, under insufficient light conditions, high competition pressure, and restricted survival space, Chinese fir shifts resource allocation toward leaf growth. suppressed within the forest, these trees compete for limited light resources. Since Leaves are the primary sites of photosynthesis, an increased leaf surface area allows trees to capture more light energy in dim environments, supplying the energy needed for survival and providing a competitive advantage in accessing sunlight. Additionally, in environments with complex forest layer structures, Chinese fir allocated more resources to branch and leaf development. Intense spatial competition within a multilayered forest affects both vertical and horizontal growth. In such cases, excessive trunk growth may be constrained by surrounding trees, limiting space expansion. However, branches and leaves can grow flexibly; when not in direct conflict with the surrounding trees, they can extend outward and occupy different canopy layers. This strategy enables trees to optimize light capture within limited space, securing an advantage in highly competitive environments.

### 4.3 Future improvements

The main purpose of this study was to explore the influence of stand spatial structure on the aboveground biomass distribution pattern of Chinese fir, so for the spatial structure parameters, whether it is the determination of structural units or the calculation of parameters, it is inseparable from the participation of tree height, crown width and DBH. In addition, in the weighted Voronoi construction, we only considered the three forest attributes: tree height, crown width and DBH, while in the actual environment, healthy trees usually have stronger ecological competitiveness and adaptability, and can accumulate biomass through vigorous photosynthesis, promote crown expansion and root extension, and thus occupy a broader ecological niche in horizontal and vertical space. In addition, different tree species will form a unique spatial distribution pattern due to their differences in biological characteristics, ecological adaptability and competitive strategies. Therefore, the health status of the trees, the type and age of the trees (Brumelis et al., 2007) will have a significant impact on the true spatial extent of the trees.

In this study, the extraction of forest parameters was based on the point cloud after terrain normalization, although the parameter extraction was more accurate, but indirectly eliminated the influence of terrain undulation on spatial structure parameters. In the forest environment, complex topography will form a variety of niches, leading to increased spatial heterogeneity of light, water and nutrients, thereby shaping the distribution pattern of forest trees. Topography will interfere with the competition of trees, and in areas with strong environmental stress, such as steep slopes or ridges, the competition between tree species may be weakened, resulting in a more uniform spatial distribution. However, in resource-rich areas such as gentle slopes or valley floors, intensified competition may lead to obvious differentiation of tree size and the formation of more complex spatial patterns. Therefore, the influence of topography on the spatial structure of forest stands will ultimately be reflected in the spatial arrangement, size differentiation and competition of forest trees, which in turn will affect the stability, productivity and ecological function of forests.

In summary, in order to truly reflect the growth of trees in the horizontal and vertical space of the forest, in addition to the tree height, crown width and DBH of the trees, we will also focus on the influence of factors such as tree health, tree type, and topography on the spatial range of forest trees in the future, so as to quantify the real situation of forest tree growth and conduct more in-depth research on the extraction of spatial structure parameters.

## 5 Conclusion

The purpose of this study was to quantify the spatial structure parameters of forests by UAV lidar, and to explore the effects of different spatial structure parameters on the biomass allocation of each component of aboveground biomass of Chinese fir. The results showed that the openness ratio (OP), competition index (UCI), forest layer index (S) and openness (K) were the key structural factors affecting the aboveground biomass allocation of Chinese fir. When the competition pressure of Chinese fir is small, the living environment is sufficient, the light reception is very open and the forest structure is relatively simple, it is conducive to the accumulation of trunk biomass of Chinese fir. When the competition pressure of Chinese fir is small, the living environment is sufficient, the light reception is open and the forest structure is complex, it is conducive to the accumulation of branch biomass of Chinese fir. When the competition pressure of Chinese fir is extremely high, the living environment is seriously insufficient, the light reception is somewhat blocked, and the forest structure is relatively complex, it is conducive to the accumulation of leaf biomass of Chinese fir. The results of this study not only reveal the survival strategy of Chinese fir in environmental change, but also provide a theoretical basis for understanding ecosystem carbon sequestration and sustainable management of Chinese fir plantations.

## Data Availability

The raw data supporting the conclusions of this article will be made available by the authors, without undue reservation.
